# Isolated Perforation of Left Coronary Cusp after Blunt Chest Trauma

**DOI:** 10.1155/2015/235629

**Published:** 2015-02-23

**Authors:** Rohit Maini, Razvan T. Dadu, Daniel Addison, Luke Cunningham, Ihab Hamzeh, Matthew Wall, Nasser Lakkis, Rashed Tabbaa

**Affiliations:** ^1^Department of Internal Medicine, Baylor College of Medicine, USA; ^2^Section of Cardiology, Baylor College of Medicine, USA; ^3^Department of Cardiothoracic Surgery, Baylor College of Medicine, USA

## Abstract

Left coronary cusp perforation is an extremely rare consequence of blunt chest trauma. A 22-year-old male presented after a motor vehicle accident with dyspnea. Transthoracic echocardiogram (TTE) and transesophageal echocardiogram (TEE) showed moderate to severe aortic regurgitation with prolapsing right coronary cusp. In the operating room he was found to have a left coronary cusp tear near the annulus and an enlarged right cusp. The patient recovered well after successful aortic valve replacement with a mechanical valve. Traumatic aortic regurgitation with left cusp perforation is serious and surgical intervention may be lifesaving if performed timely.

## 1. Introduction

Left coronary cusp perforation is an extremely rare consequence of blunt chest trauma but still an important cause of severe aortic insufficiency with serious complications. This is a case of a young gentleman with this uncommon disease process.

## 2. Case Presentation

**Figure 1:**
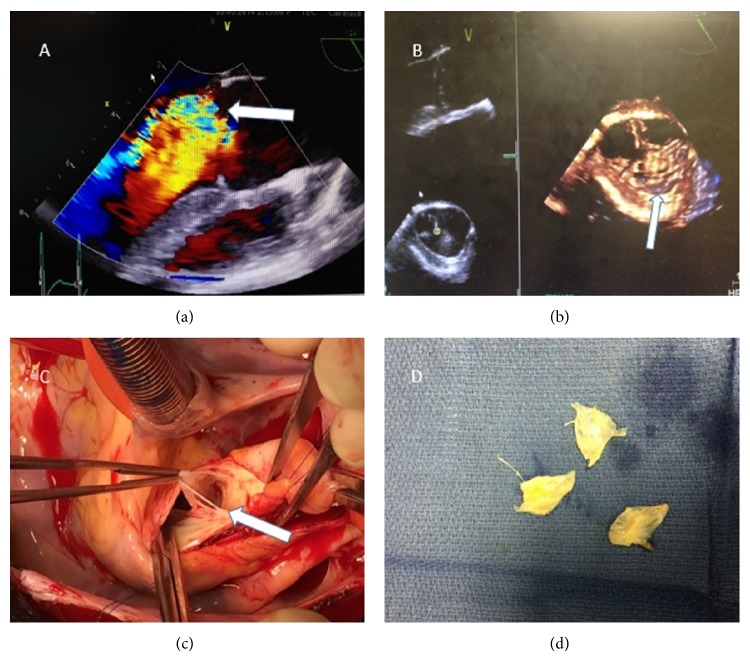
(a) TEE with color Doppler showing moderate to severe aortic regurgitation. (b) 3D by TEE showing prolapsed right coronary cusp. (c) Intraoperative image showing perforation of the left coronary cusp. (d) Pathologic specimen showing the three cusps.

A 22-year-old male presented to the emergency room following a motor vehicle accident with an open right ankle, evidence of a femoral fracture, and shortness of breath. His past medical history was not significant for any medical conditions. He denied any drug abuse or smoking as well as any family history of heart disease. On initial exam, he was awake and afebrile; his blood pressure was 124/68 mm Hg and heart rate was 140 beats per minute, with an O_2_ saturation 100% on room air. Cardiovascular exam was notable for abnormal vital signs with a diastolic click heard best at the right second intercostal space. Cardiology was consulted on hospital day 6 due to runs of asymptomatic supraventricular tachycardia. Following the appreciation of a diastolic murmur, a TTE was performed showing moderate to severe aortic regurgitation by color Doppler (Figure 1) with a borderline normal left ventricular end-diastolic diameter concerning acute aortic regurgitation. A TEE was then performed to further delineate the aortic valve pathology, revealing an eccentric severe aortic regurgitation jet directed toward the anterior mitral valve leaflet (Figure 1) along with a prolapsing right coronary cusp concerning traumatic avulsion. The hospital course was then complicated by development of* Clostridium difficile* colitis for which patient received treatment. Following this, on hospital day 23 the patient underwent open heart surgery where he was found to have a tricuspid aortic valve with a left coronary cusp tear near the annulus as well as an enlarged prolapsing right coronary cusp. The valve was replaced with a 21 mm mechanical St. Jude Regent valve. Pathology revealed myxoid degeneration of the aortic valve leaflets without evidence of prior endocarditis or calcifications. The patient recovered without sequelae and is doing well at 2-month follow-up.

## 3. Discussion

Coronary cusp perforation after MVA is a rare and potentially fatal lesion that can present with signs/symptoms at any time within the first several weeks after injury. Several previous reports have described right or noncoronary cusp perforation after MVA [1–6]. The first reported case of acute aortic valve tear in 1830 occurred following autopsy after a traffic accident. Afterwards, no more than one hundred cases had been reported worldwide through 2002 [2]. The spectrum of trauma type, presenting age, time from injury, and symptoms were significantly variable, ranging from dyspnea or an isolated murmur to hemodynamic instability with profound decompensated heart failure [3, 5, 6]. Patients who present late after trauma are more likely to have a tear in the cusp which progresses over time to cause symptoms.

The pathophysiology of isolated cusp perforation from blunt chest trauma remains unknown. However the most likely explanation appears to involve the compressive force applied during early diastole in the setting of trauma causing a sudden increase in intrathoracic pressure when the aortic valve is closed and the transvalvular gradient is maximal. Concurrently, the mechanism of aortic regurgitation likely involves injury to the aortic root or isthmus, as this is the most vulnerable site of the thoracic aorta [7]. When cusp isolated pathology is present, the noncoronary cusp is preferentially affected. The mechanism is unclear but appears to involve direct damage to the valve apparatus or prolapse of the subadjacent valve cusp due to rupture of the ascending aorta. Tear or avulsion from the annulus of one aortic valve cusp is the most frequently observed valve lesion [4] likely due to the impact of a hard surface with limited space defined by the chest. Therefore, blood within the aorta would be displaced leading to rupture of the ascending aorta or valve apparatus. Rupture of the valve apparatus is more likely during diastole when the left ventricular pressure is low as compared to systole when the left ventricular counterpressure can protect the aortic valves.

In contrast, the left coronary cusp appears to be least affected in blunt trauma presentations. The mechanism by which the left cusp is relatively protected likely involves pressure off-loading from diastolic coronary flow. Furthermore, the left coronary cusp is a more posterior structure compared to the right or noncoronary cusp, serving as a natural anatomic shield from the highest forces [1].

Our case is one of the few cases reported in the literature. Pathologic examination confirmed myxoid degeneration which predisposed to cusp perforation [8]. Myxomatous degeneration macroscopically results in a thin region in each leaflet microscopically due to ongoing substitution of the collagen by mucopolysaccharides in the spongiosa leading to progressive weakness of the band [8].

Multiple approaches to management of traumatic cusp rupture have been considered [3–6]. A conservative approach of monitoring signs of clinical or hemodynamic deterioration has been proposed. This is based on the potential relative tolerability of the insufficiency in some patients and/or on the consideration of high-operative risks. However, as noted in multiple reports, the onset of symptoms or hemodynamic compromise appears to portend higher long-term morbidity. Once a patient becomes symptomatic, there is likely some degree of irreversible LV dysfunction, which may also influence surgical outcome(s) as well as long-term survival [5]. In light of this, a more aggressive approach with early surgical intervention seems to represent the optimal treatment strategy. Of the case reports reviewed, only three cases were treated with repair, reconstruction, or valvuloplasty over replacement likely due to the level of valve injury. Aortic valve replacement appears to represent a more effective approach [1, 3–6].

## 4. Conclusion

Traumatic coronary cusp perforation is a rare but important cause of severe aortic insufficiency. Clinical presentation is variable with little regard for the time from initial trauma. Dyspnea in association with a new diastolic murmur and widened pulse pressure is the most common presentation, but any abnormal cardiac exam after blunt trauma should raise suspicion. The potential for rapid deterioration due to acute aortic regurgitation makes prompt diagnosis of valve rupture paramount. Given the high risk for significant morbidity and mortality, prompt surgical intervention should be considered in all cases.
